# Supplementary figures and images for: Using the National Trauma Data Bank (NTDB) and machine learning to predict trauma patient mortality at admission
Source: PLoS One. 2020 Nov 17;15(11):e0242166. doi: 10.1371/journal.pone.0242166 (PMC7671512; doi:10.1371/journal.pone.0242166)

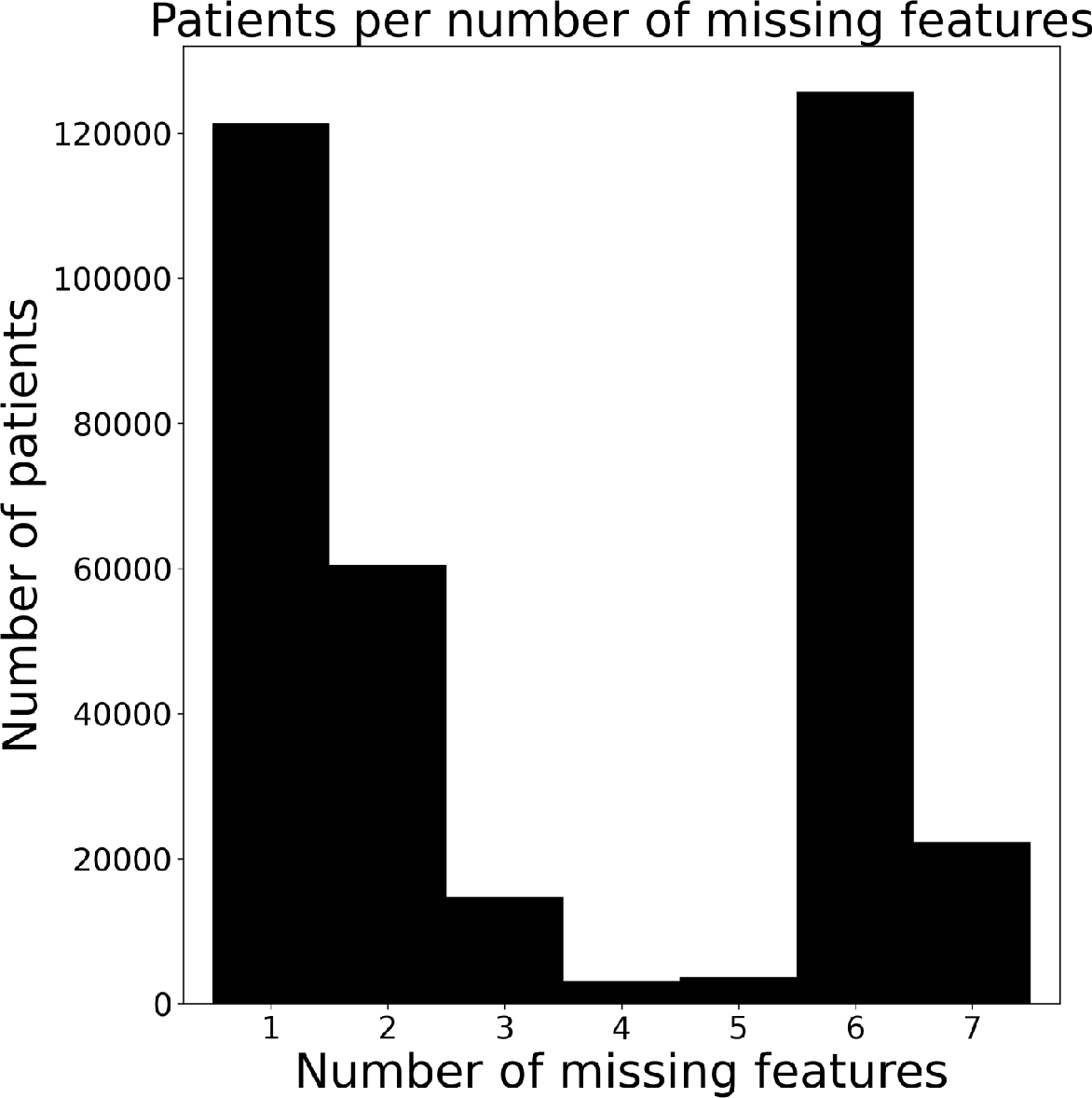

Supplement: S1 Fig — The distribution is bimodal, suggesting that including patients with a maximum of 2 missing features is the appropriate threshold for inclusion. (TIF) [file pone.0242166.s001.tif]

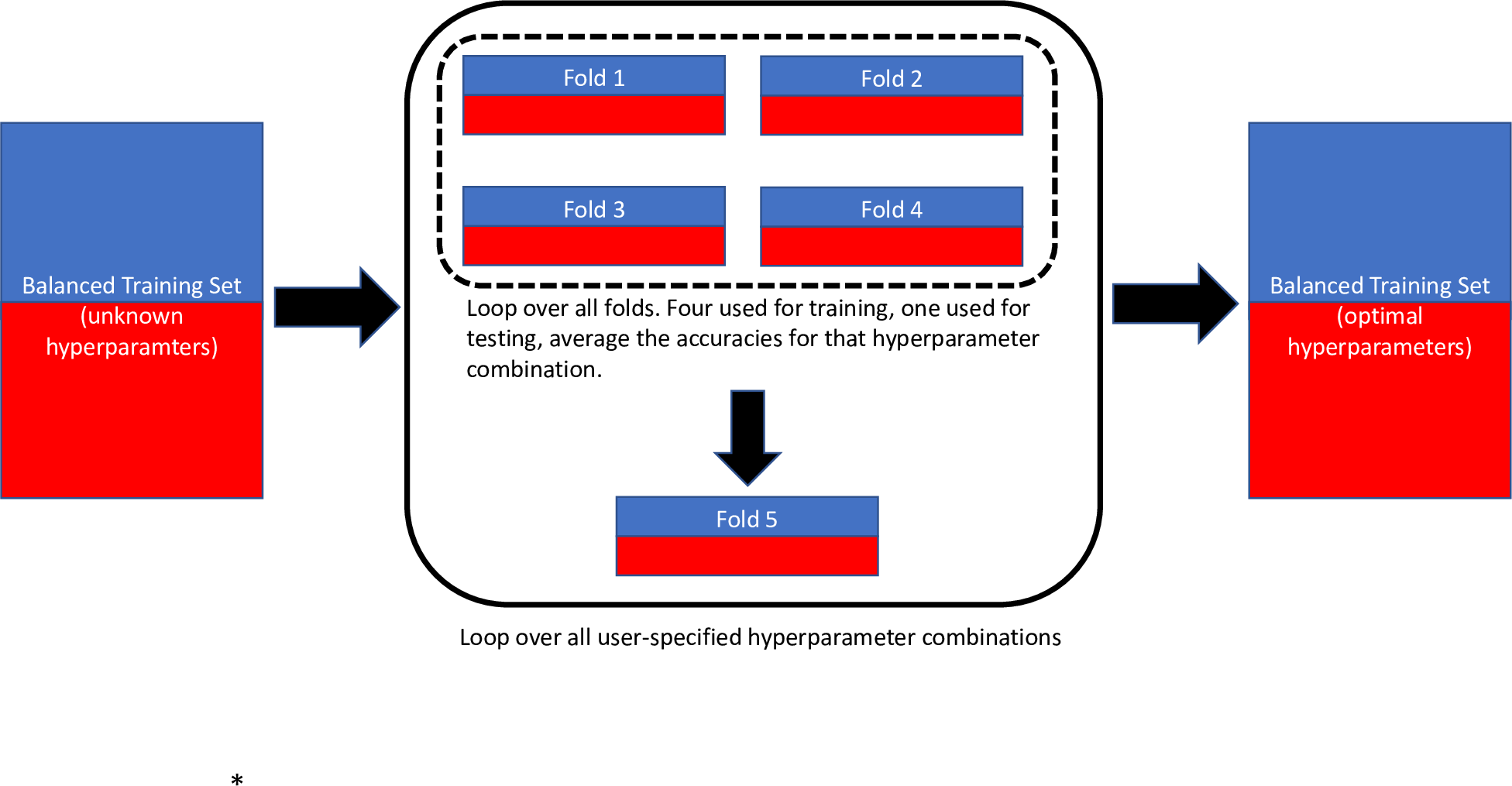

Supplement: S2 Fig — Importantly, the test set was withheld during the grid-search cross validation process allowing it to remain a fair metric for evaluating performance on new data. (TIF) [file pone.0242166.s002.tif]

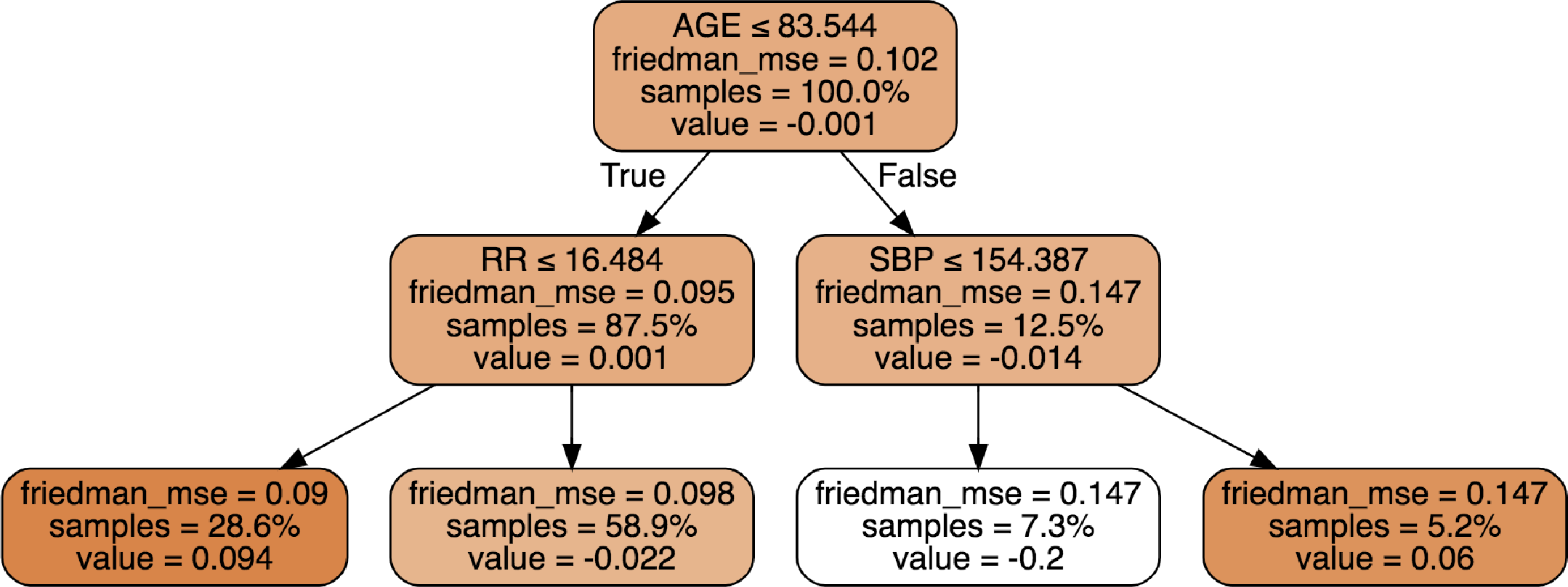

Supplement: S3 Fig — Variable thresholds, Friedman mean squared errors [19], percentage of training samples passed through each node, and log odds ratios are all present. (TIF) [file pone.0242166.s003.tif]
