# Supplementary material for: Using the National Trauma Data Bank (NTDB) and machine learning to predict trauma patient mortality at admission
Source: PLoS One. 2020 Nov 17;15(11):e0242166. doi: 10.1371/journal.pone.0242166 (PMC7671512; doi:10.1371/journal.pone.0242166)
Supplement: S1 Table — (DOCX) [file pone.0242166.s004.docx]

**S1 Table Table of all features used to make predictions**

| **32 features** | **8 features** |
| --- | --- |
| GCSTOT | GCSTOT |
| Age  HR  SBP  TEMP  GENDER  RR  SaO2  Advanced Directive Limiting Care  Alcohol Use Disorder  Attention Deficit Disorder  Bleeding Disorder  Cerebrovascular Accident  Chronic Obstructive Pulmonary Disease  Chronic renal failure  Cirrhosis  Congenital Anomalies  Congestive Heart Failure  Current Smoker  Chemotherapy for cancer  Dementia  Diabetes Mellitus  Disseminated Cancer  Drug use disorder  Functionally Dependent  Peripheral Vascular Disease  History of Angina  History of Myocardial infarction  Hypertension  Major Psychiatric Illness  Prematurity  Steroid Use | Age  HR  SBP  TEMP  GENDER  RR  SaO2 |
