# Supplementary material for: Using the National Trauma Data Bank (NTDB) and machine learning to predict trauma patient mortality at admission
Source: PLoS One. 2020 Nov 17;15(11):e0242166. doi: 10.1371/journal.pone.0242166 (PMC7671512; doi:10.1371/journal.pone.0242166)
Supplement: S1 File — (DOCX) [file pone.0242166.s005.docx]

**5. Supplemental Section**

**5.1 Gradient Boosting Classifier**

Many different machine learning algorithms exist for identifying trends and hidden patterns in a large, high dimensional dataset like the NTDB. Since the NTDB contained labeled data (i.e., *survived* and *deceased*) we focused on supervised learning approaches. This class of machine learning algorithms (e.g., support vector machine, neural network, random forest, etc.) learn a function that maps a high dimensional input to an output. Here, we choose a gradient boosting classifier because it was found to be the most accurate when compared with several other models; see supplemental Table S2. The gradient boosting algorithm utilizes a stage-wise addition of weak learners, small decision trees (stumps), and averages the prediction of each of them to make a final decision on the individual patient. During the training process, subsequent learners are trained with an added weight on the individual samples that have been classified incorrectly, as shown in Fig. 1D.

**Table S2 Performance comparison of various supervised machine learning models.**

| **Model** | **ROC-AUC** |
| --- | --- |
| Gradient Boosting Classifier | 0.918 |
| Neural Network  Decision Tree Classifier  Adaboost Classifier  Random Forest | 0.910  0.817  0.907  0.896 |

Most machine learning algorithms contain hyperparameters, which are parameters that must be specified prior to the start of the training process. The *k*-fold grid-search cross validation method was used to determine the optimal set of hyperparameters (number of weak learners, learning rate, the fraction of the data used for training by each learner, and the number of features to consider at each split point) for the gradient boosting classifier. The training set was temporarily subdivided into 5 folds (*k* = 5) and the model was trained with 4 of the folds and evaluated on the 5^th^ fold. The accuracy was recorded and repeated, each time changing which fold the model was evaluated on, and the final accuracy was taken as the average of each of these. This process was repeated for each combination of hyperparameters and whichever combination produced the smallest error rate was taken as the optimal set. The validation set was returned to the training set and the model was trained and evaluated on the test set with the parameters determined to be the most optimal by the grid-search cross validation method. Note that the test data was not used during the *k*-fold grid search or the model training. A visual description of this process is shown in Supplemental Fig. S2 and an example of a single weak learner from the trained model is shown in Supplemental Fig. S3.

**Fig. S2 The 5-fold grid-search cross validation method for selecting the approximately optimal hyperparameters.** Importantly, the test set was withheld during the grid-search cross validation process allowing it to remain a fair metric for evaluating performance on new data.

**Fig. S3 A single weak learner randomly chosen from the trained gradient boosting ensemble of weak learners.** Variable thresholds, Friedman mean squared errors [19], percentage of training samples passed through each node, and log odds ratios are all present.

**5.2 Global Feature Dependencies - Partial dependence**

Once a model has been trained and tested, partial dependence curves on each feature can be used to garner a greater understanding of how the model depends upon each feature. Partial dependence is the marginal effect that a feature has on the model’s prediction and can be used to show the form of the dependence (linear, exponential, monotonous, etc.) and whether the feature affects the prediction positively or negatively. The partial dependence function is defined as

|  | $f\left( x_{i} \right)= \int f\left( x_{i},x_{R} \right)dx_{R},$ | (2) |
| --- | --- | --- |

where *f*(*x*) is the trained model, *i* is the feature of interest, *x_F_* is the vector of values for feature *F*, *R* is the set of all remaining features, and *x_R_* is the dependence of *f*(*x*) on the remaining features. Since the partial dependence curves are constructed by marginalizing over all remaining features, the resulting model is a function of feature *i* only. We can approximate this integral by discretizing equation (2), e.g.,

|  | $f\left( x_{F} \right)=\frac{1}{n} \sum_{i=1}^{n} f\left( x_{i},x_{R}^{(i)} \right),$ | (3) |
| --- | --- | --- |

where *n* is the number of samples in the training set. This is a sensitivity analysis averaged over all test set examples. Since this method marginalizes over all features, a requirement is that feature *i* must be uncorrelated with the features in R. All Pearson correlation coefficients were < 0.6, verifying this assumption.

**5.3 Patient Specific Feature Dependencies - Shapley Additive Explanations (SHAP)**

While partial dependence curves provide a global view of the effect of each feature, Shapley Additive Explanations (SHAP) is a method for calculating the relative importance of each feature on a single prediction (patient)[5, 24-25]. The method was largely inspired by Lloyd Shapley’s work in cooperative game theory, where individual players are rewarded based upon their contributions in a collaborative game [26]. When applied analogously to the prediction of a model, the SHAP value measures how much each feature contributes to its prediction. This feature importance tool has the advantage of accounting for local patient-to-patient variations, which is important since a significant factor for one patient may not be for another. By contrast, global feature importance metrics, such as partial dependence curves, average out variations across the entire population.

Analogous to linear regression, the SHAP method locally approximates the function, *f(x)*, by a linear combination of the features each weighted by a SHAP value (ϕ), i.e., its contribution to the model prediction. Intuitively, the SHAP value of a feature is the difference in the prediction of the model with and without the feature present. While the model requires 8 inputs to run in general, it can be allowed to run by computing a weighted average of all nodes that depend upon the missing feature. The SHAP value computation of a feature, *i*, requires averaging the output of the model, *f*, for all possible subsets of features with and without *i*. Formally, the SHAP value is computed with the formula,

|  | $\varphi_{i}\left( x \right)= \sum_{S\subseteq N\backslash\{i\}} \left( \frac{\left\vert S \right\vert\left( M-\left\vert S \right\vert-1 \right)!}{M!} \right)*\left( f_{x}\left( S \cup\left\{ i \right\} \right)-f_{x}\left( S \right) \right),$ | (4) |
| --- | --- | --- |

where *S* is the set of features being considered, *|S|* is the cardinality of *S*, *M* is the total number of features, *f_x_* is the prediction made by the model, *N* is the set of all input features, and $\varphi_{i}(x)$ is the shapley value of the *i*^th^ feature of patient *x* [24]. Intuitively, the second term in equation (4) represents the importance of a feature as the difference in the prediction with and without that feature present, weighted by the first term. More information on this computation can be found in the original work by Lundburg et al. [5].

**5.4 Iterative Imputation**

We used the IterativeImputer class from the scikit-learn library to impute missing data in patients missing 2 or fewer features [27]. Missing features are modeled as functions of present features and a ridge linear regression model is trained to predict the missing value. At a single step in the iteration, a single feature is treated as the missing output while the remaining features are the input. This is repeated for each feature in a single round, and then iterated for 50 rounds. In the rare instances that this imputation led to unphysical values (e.g., age < 0), we simply imputed the value with a nearby physical value. This is superior to simply imputing the missing value with the mean of the present features as it imputes the average over many approximations of possible values. Furthermore, simple imputation significantly model variance leading to lower generalizability.
